# Early-stage cervical cancer diagnosis based on an ultra-sensitive electrochemical DNA nanobiosensor for HPV-18 detection in real samples

**DOI:** 10.1186/s12951-020-0577-9

**Published:** 2020-01-13

**Authors:** Pegah Mahmoodi, Majid Rezayi, Elisa Rasouli, Amir Avan, Mehrdad Gholami, Majid Ghayour Mobarhan, Ehsan Karimi, Yatima Alias

**Affiliations:** 1 Department of Biology, Mashhad Branch, Islamic Azad University, Mashhad, Iran; 2 Medical Toxicology Research Center, Mashhad University of Medical Sciences, Mashhad, Iran; 3 Metabolic Syndrome Research Center, School of Medicine, Mashhad University of Medical Sciences, Mashhad, Iran; 4 Department of Medical Biotechnology and Nanotechnology, School of Medicine, Mashhad University of Medical Sciences, Mashhad, Iran; 5 Nanotechnology & Catalysis Research Centre, Institute of Postgraduate Studies, University Malaya, 50603 Kuala Lumpur, Malaysia; 6 Department of Chemistry, Marvdasht Branch, Islamic Azad University, P.O. Box 465, Marvdasht, Iran; 7 Department of Chemistry, University of Malaya, 50603 Kuala Lumpur, Malaysia

**Keywords:** Electrochemical, Biosensor, DNA, HPV-18, Cervical cancer

## Abstract

**Background:**

In several years ago, infection with human papillomaviruses (HPVs), have been prevalent in the worlds especially HPV type 18, can lead to cervical cancer. Therefore, rapid, accurate, and early diagnosis of HPV for successful treatment is essential. The present study describes the development of a selective and sensitive electrochemical biosensor base on DNA, for early detection of HPV-18. For this purpose, a nanocomposite of reduced graphene oxide (rGO) and multiwalled carbon nanotubes (MWCNTs) were electrodeposited on a screen-printed carbon electrode (SPCE). Then, Au nanoparticles (AuNPs) were dropped on a modified SPCE. Subsequently, single strand DNA (ssDNA) probe was immobilized on the modified electrode. The link attached between AuNPs and probe ssDNA provided by l-cysteine via functionalizing AuNPs (Cys-AuNPs). The differential pulse voltammetry (DPV) assay was also used to electrochemical measurement. The measurement was based on the oxidation signals of anthraquninone-2-sulfonic acid monohydrate sodium salt (AQMS) before and after hybridization between the probe and target DNA.

**Results:**

The calibration curve showed a linear range between 0.01 fM to 0.01 nM with a limit of detection 0.05 fM. The results showed that the optimum concentration for DNA probe was 5 µM. The good performance of the proposed biosensor was achieved through hybridization of DNA probe-modified SPCE with extracted DNA from clinical samples.

**Conclusions:**

According to the investigated results, this biosensor can be introduced as a proprietary, accurate, sensitive, and rapid diagnostic method of HPV 18 in the polymerase chain reaction (PCR) of real samples.

## Introduction

Several studies demonstrated that HPV is commonly spread by sexual activity. HPVs are classified according to their risk of cancer infection into two groups (high risk and low-risk). Among the high-risk HPVs, the most dangerous types are HPV-16 and HPV-18 that are responsible for almost more than half of cervical cancers. Also, the high-risk HPVs are responsible for 36% of penile cancers, 51% of vulvar cancer, 63% of oropharyngeal carcinomas, 64% of vaginal cancers, 93% of anal cancers and 96% of cervical cancers [1]. Double-stranded DNA genomes of HPVs encode eight genes, which among them, E6 and E7 by having transforming properties, are necessary for malignant conversion [2]. Traditional methods for detection of HPVs are Pap smear and visual inspection with acetic acid. These methods have their own limitations such as poor specificity and sensitivity [3]. However, due to recent advances in molecular methods, this method is used to detect and also identify the types of HPV. For example, COBAS^®^4800, digene HC2 High-Risk HPV DNA, linear array^®^, PapilloCheck^®^, INNO-LiPA, clinical arrays^®^HPV, CLART^®^human papillomavirus 2 and others are the assays that showed a high sensitivity for the diagnosis of HPVs and pre-cancerous lesions. Nevertheless, the methods mentioned have limitations such as high cost, long response time, requirment of trained personnel as well as advanced equipment and the need for PCR products [4, 5]. Therefore, considering many disadvantages of the existing diagnostic methods, the development of an appropriate diagnostic method for identifying HPV is essential. In recent years, DNA biosensors are introduced as a new appropriate device for rapid, simple, economical, sensitive, specific and early detection of pathogens [5–7]. They are diagnostic devices that are designed for detection of DNA target among million-fold excess of non-target species, by hybridized complementary DNA probes immobilized on the surface of various types of electrodes [8]. Until now, different biosensors for HPVs assays were reported, but electrochemical biosensors have more advantageous due to their portability, cost-effectiveness, small sample volume, small size, and ease of use [9–11]. In order to improve the performance of the biosensor, various materials are used to modify the surface of the electrode. Research conducted in recent years shows that rGO is a suitable candidate for improving the performance of electrochemical activities in biosensors. The structure of rGO honeycomb produces significant either chemical or physical properties, such as high surface area, high electrical conductivity and high chemical stability. Furthermore, the introduction of MWCNTs into the matrix of rGO-based materials not only improves electron transfer but also provides more porous structure, which is a very effective way to increase sensitivity and improve biosensor performance [12–14]. Moreover, recent advances in biosensors indicate that the use of Au nanoparticles increases electrical conductivity and provides the immobilization passages of DNA on the electrode surface [15]. Many of the amino acids have special properties that make it possible to use as an effective ingredient to immobilize biomolecules on the surface of the electrode. They have special groups that are capable of binding with biomolecules. This improves the immobilization of DNA [16]. The amino acid l-Cysteine is an effective ingredient for electrode surface modification. It has a thiol group, which can bond strongly to Ag or Au electrodes through bonding of amino groups to DNA [17–19]. Due to the high prevalence of HPV and the lack of appropriate diagnostic methods in the primary stages of infection, in this investigation a sensitive and accurate diagnostic method for the specific identification of HPV 18 is introduced. It is well known that the development of the novel sensing nanomaterials such as rGO, MWCNT and AuNPs has been proven as an effective method to fabricate electrochemical DNA biosensors for diagnosis of cancer markers. To the best of our knowledge, this is the first report of functionalized electrochemical DNA-biosensor for HPV-18 detection based on rGO-MWCNT/l-Cys-AuNPs nanocomposite in the real samples.

## Materials and method

### Reagents and materials

All chemicals and reagents were of analytical reagent grade. All aqueous solutions were prepared using deionized water with resistivity of ∼ 18 MΩ cm. Anthraquninone-2-sulfonic acid monohydrate sodium salt (AQMS), l-cysteine and sodium chloride were purchased from Merck (Germany). Multi-walled carbon Nano tube (MWCNT), gold nanoparticles (AuNPs) and sulfuric acid were purchased from Sigma Aldrich (USA). The potassium permanganate and graphite flakes were purchased from R & M Chemicals (U.K). The applied solvents throughout the experiments such as ethanol, sulphuric acid, phosphoric acid, hydrochloric acid, and hydrogen peroxide were in analytical grade and purchased from Merck (Germany). All single-stranded DNA (ssDNA) was purchased from TAG Copenhagen Company (Denmark). Screen-printed carbon electrodes were supplied by DropSens (Asturias).

### Apparatus

The electrochemical measurement was carried out with a Galvanostat–Potentiostat; (Autolab PG-STAT-204). The voltammetry measurements were performed on a three-electrode system. The working electrode was a screen printed carbon electrode (4 mm OD). Ag/AgCl, 3 M KCl, RE-5B (7.5 cm × 6 mm OD) and platinum wire electrode (BASi^®^, USA) were used as a reference and auxiliary electrodes, respectively. Electrochemical impedance spectroscopy (EIS) analysis was conducted using a Galvanostat-Potentiostat, Autolab 302 N with FRA2 impedance module controlled by Nova 1.11 software, Metrohm, Switzerland. The ultrasonic homogenizer (Sonopuls, HD 3200) was applied throughout the experiments for homogenizing and breakdown of nanoparticles. All hybridization experiments were carried out in a water bath (Memmert, WNB 14).

### Human papillomavirus synthetic DNA oligonucleotides

All oligonucleotide stock solutions were diluted with phosphate-buffer (0.05 M, pH = 7.0) and kept frozen at − 20 °C when not used. The following three oligonucleotide sequences were used in this study (Table 1).

**Table 1:** The oligonucleotide-based DNA sequences used in the current study

DNA	Base sequences
DNA probe	5′-CCG GTG CAG CAT CC-3′
Complementary DNA	5′-GGA TGC TGC ACC GG-3′
One mismatch DNA	5′-GCT AGA GGT GTA TG-3′
Three mismatch DNA	5′-GCT AGA GAT GCA TG-3′
Non-complementary DNA	5′-CAC ATC CAC CCG CA-3′

### DNA extraction, PCR method and HPV genotyping

For extracting DNA from endocervical of patients infected with HPV 18, the High Pure Viral Nucleic Acid Kit (Roche Life Science, Mannheim Germany) was used. For HPV testing, a DNA-based liquid-crystal display (LCD)-array kit (Chipron GmbH, Berlin, Germany) was applied as described previously [20, 21]. The PCR program is presented in Table 2. In total, the PCR was accomplished in Veriti^®^ 96-Well Thermal Cycler (Thermo Fisher Scientific; Applied Biosystems, USA).

**Table 2:** The PCR cycling conditions

Steps	Temp. (°C)	Time (min)	Cycles
Initial denaturation	95	10	1
Denaturation	94	1	42
Annealing	45	1.5	
Extension	72	1.5	
Final extension	72	3	1

The positive control had DNA from a HPV 18 case while the negative control had no DNA. According to the manufacturer’s protocol (Chipron GmbH) PCR, hybridization, labeling, and staining were carried out. The slide was scanned by SlideReader Software v2.0 (Chipron GmbH), followed by gel electrophoresis.

### Preparation of the modified SPCE

**Fig. 1:**
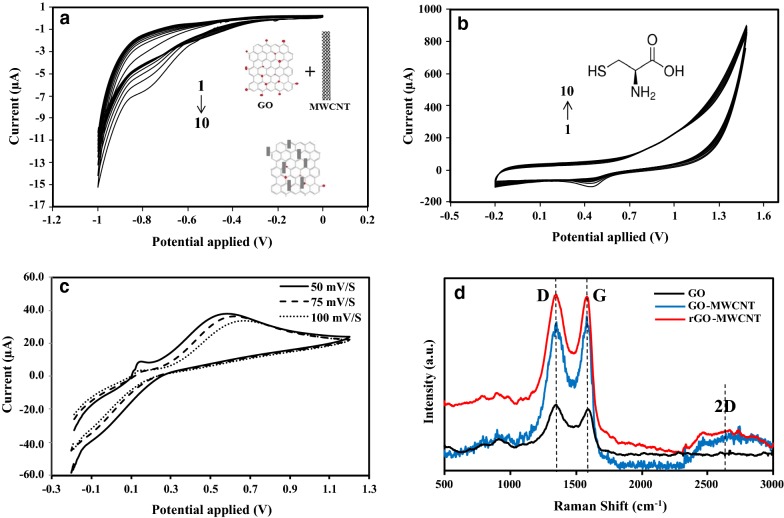
Cyclic voltammograms of electrodeposition of (**a**) rGO-MWCNT nanocompoite on a bare SPCE (in 0.2 M PBS, pH 6.5 and Scan rate: 50 mVs^−1^), **b** of l-cysteine (10 mM) on a modified SPCE (in 0.1 M PBS, pH 7.4; Scan rate: 50 mVs^−1^), **c** the effect of different scan rates on electrodeposition CV curves of rGO-MWCNT nanocompoite in 1 × 10^−3^ M [Fe(CN)6]^−3^/[Fe(CN)6]^−4^ (1:1) containing 0.1 M KCl solution and **d** Raman spectra of GO, GO with MWCNT and rGO-MWCNT nanocompoite

For this purpose, DI water was used for washing the bare SPCE and then the electrode was electrochemical polished using cyclic voltammetry (CV) in 0.1 M H_2_SO_4,_ ranging from (0.5 to 1.5 V) with a scan rate of 50 mVs^−1^. The modified Hummer’s method was applied for the synthesis of GO based on our previous work. Prior to deposition, the solution of GO and MWCNT was prepared by dispersing 5.0 mg of GO and 11.0 mg of MWCNT into 0.2 M PBS, pH 6.5 after 2 h of sonication. Electrodeposition of rGO-MWCNT nanocomposite on a SPCE in a one-step approach was performed by CV method through scanning between (− 1.0 and 0 V) at a scan rate of 50 mVs^−1^ until 10 cycles (Fig. 1a).

The AuNP colloidal solution was made by dissolving 1 mg of the powder in 300 µl mixture of DI water and ethanol (1:1) followed by 1 h sonication and then drop cast onto the surface of SPCE. l-cysteine (10 mM) in 0.1 M PBS, pH 7.4 was electrochemically deposited on the modified SPCE through CV scanning between (−0.2 and 1.5 V); 10 cycles; scan rate of 50 mV s^−1^ (Fig. 1b).

As illustrated in Fig. 1a, MWCNT was deposited on the SPCE in the potential range of − 0.6 to − 0.8 V. Electrodeposition of GO on SPCE in the potential range from − 0.8 to − 1.0 V led to slightly reduce oxygen functional groups which resulted to increase the access to carbon groups and its efficiency. Also, according to Fig. 1a, it can be seen that in with increasing the number of cycles, the cathodic peak current increased due to the increment in the amount of rGO-MWCNTs deposited on the surface of SPCE during each cycle, and enhance the electrical conductivity [12, 22, 23].

**Scheme 1:**
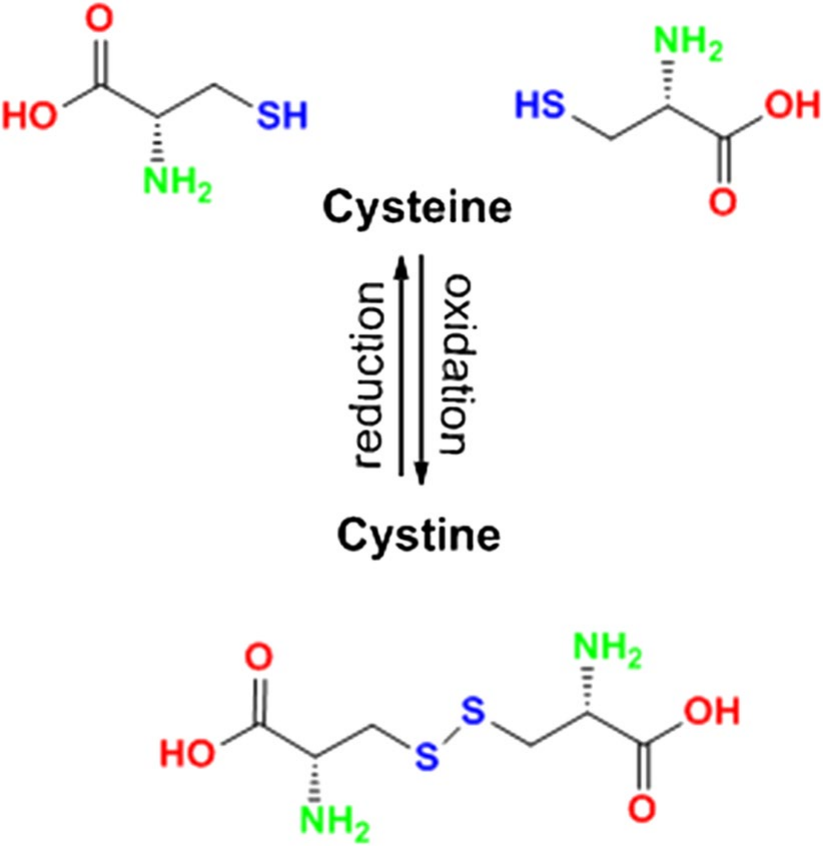
Immobilization mechanism for l-cysteine onto SPCE

Figure 1b shows the CV curves of electrodeposition of l-cysteine in the potential range of − 0.2 V to + 1.5 V with a scan rate of 50 mV s^−1^. A reduction peak occurs at 0.43 V during the reduction scan and related to the SH group. The reduction peak current increased with the increment of the cycle number. The reaction indicated in Scheme 1, occurs during the electrodeposition process [24–26]. The electrodeposition of rGO-MWCNT on SPCE at variable scan rates revealed a capacitive improvement with decreasing the rate of scans from 100 to 50 mV/S (see Fig. 1c; 314 < 316 < 324 µF). The capacitance was calculated based on the following equations [27]:

1 $$C^{\prime} = {I \mathord{\left/ {\vphantom {I {(\Delta V/\Delta t)}}} \right. \kern-0pt} {(\Delta V/\Delta t)}}$$

2 $$C = {{C^{\prime}} \mathord{\left/ {\vphantom {{C^{\prime}} {Area}}} \right. \kern-0pt} {Area}}\,or\,{{C^{\prime}} \mathord{\left/ {\vphantom {{C^{\prime}} {mass}}} \right. \kern-0pt} {mass}}$$

where, I and ∆V/∆t are the measured current and voltage scan rate, respectively.

As illustrated, the electrodeposition of rGO-MWCNT with a scan rate of 50 mV/S on SPCE has a large integrated CV area indicating of higher capacitance (324 µF). Figure 1d report the Raman spectra of synthesized GO, GO with MWCNT and rGO-MWCNT nanocomposite which shows the prominent peaks of D band at ~ 1358 cm^−1^ and G band at ~ 1587 cm^−1^, respectively. It is well known that, the D band arising from structural disorder, while G band arising from in-plane C–C bond stretches [27–30]. The 2D mode is also contribute to the double resonance of sp^2^ carbon domains and occurs at 2550 to 2800 cm^−1^. As seen in Fig. 1d the existence of MWCNT demonstrated a broad 2D peak. The 2D band in the Raman spectrum of the rGO-MWCNT becomes more intense, indicating that the graphene sheets are well separated due to reducing the carboxylic groups after electrodeposition process. The intensity ratio of D band to G band (I_D_/I_G_) is taken as a measure of disorder. It is obvious from Fig. 1d that the ID/IG for the rGO-MWCNT nanocomposite is lower than GO-MWCNT which indicates the disordered carbon structure. The ratio of 2D to D + G in GO-MWCNT and rGO-MWCNT is higher than GO due to its broad 2D peak. The Raman spectroscopy results revealed that the resulting morphology well incorporates both rGO and MWCNT in the nanocomposite during the electrodeposition process.

### Immobilization of HPV-18 DNA probe

In order to immobilize ssDNA probe on the modified SPCE by the dip-coating method, the electrode was placed in the solution of HPV-18 DNA (1 µM) in 0.05 M PBS, pH 7.0 for 24 h at 25 °C. Then, to remove the unbound oligonucleotides, the electrode was washed with 0.05 M PBS, pH 7.0. The development of DNA microarrays was provided by bounding the negatively charged phosphate backbone of the DNA probes with a positively charged from amine groups of l-cysteine. The stability of the probe through the ionic attraction achieved via applying the potential during immobilization process [31, 32].

### DNA hybridization

Most of electrochemical DNA biosensor was developed based on separate processing of DNA hybridization and indicator intercalation. In the current study, the proposed DNA biosensor was preformed according to the one-step of DNA hybridization and indicator intercalation detection.

**Fig. 2:**
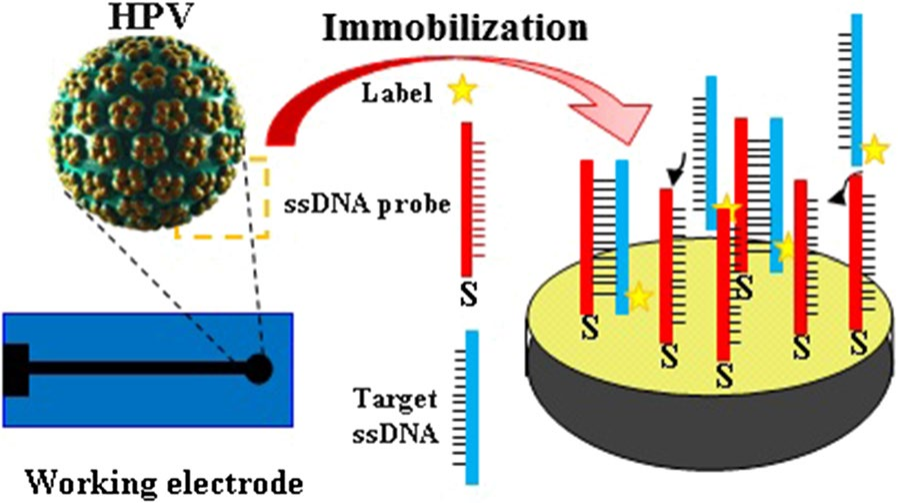
The proposed HPV 18 DNA biosensor design based on the immobilization of the ssDNA probe, DNA hybridization, and indicator intercalation detection

Hybridization and labeling event was performed by soaking the immobilized HPV18 DNA probe into the solution containing the complementary DNA (cDNA) and AQMS in 0.05 M PBS (pH 7.0) for 15 min at 42 °C. The 0.05 M PBS pH 7.0 was used to remove the nonhybridized oligonucleotides. For the interaction of HPV18 probe with noncomplementary DNA, the same process was applied. In order to the hybridization of the genomic DNA with HPV18 DNA probe on the modified SPCE, the extracted DNA from the real sample was denatured by heating in a water bath (95 °C) for 10 min and then placed at room temperature to obtain denatured ss-DNA. The SPCE was washed with 0.05 M PBS, pH 7.0 to remove the nonhybridized sequences. The schematic process of designing the proposed HPV 18 DNA biosensor including the immobilization and hybridization of DNA oligomers is illustrated in Fig. 2.

### Electrochemical measurements

The Electrochemical analysis were studied by DPV method. The electrochemical measurement of ssDNA probes immobilized l-cys-AuNPs composite SPCE with AQMS immobilized in cDNA was performed in 0.05 M PBS, pH 7.0, under the potential sweep between − 0.7 and − 0.2 V with a scan rate of 50 mV s^−1^.

## Results and discussion

### Preliminary studies

In this study, the hybridization of DNA probe and target DNA was studied by UV–Vis spectroscopy and electrochemical oxidation of AQMS. One of the common methods for detection of nucleic acid is an absorbance measurement via UV–Vis spectroscopy. AQMS as an anionic redox intercalator usually used for determination of DNA hybridization [33, 34]. In this study, the mechanism of HPV 18 DNA biosensor based on DNA hybridization sensing was studied through the AQMS electrostatic adsorbtion onto the sensing element of double-stranded DNA.

**Fig. 3:**
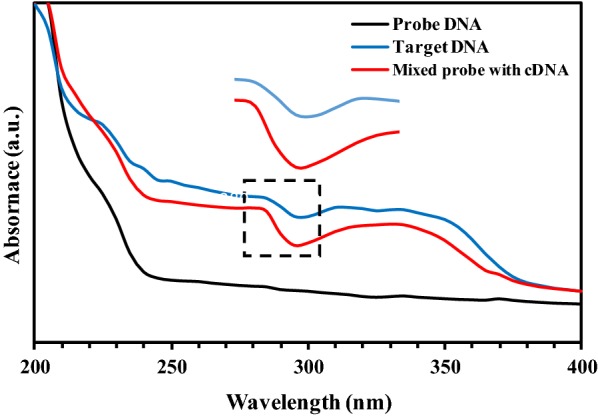
UV-Vis absorbance spectra of DNA probe, cDNA and their hybridization containing 1 mM AQMS in 0.05 M PBS at pH 7.0

For the UV–Vis study, the modified SPCE was soaked in a solution contained AQMS redox-active and cDNA. After a period of time, the UV–Vis technique was applied to the modified SPCE to investigate the surface species. As illustrated in Fig. 3, the AQMS showed a strong absorption spectra at 295 nm as well as a weakly absorption spectra at 225 nm of DNA associated with n → π* and π → π* transition state. A high absorbance signal with a slightly red-shift of spectra would be observed from the dsDNA modified electrode. This is confirmed by the action of AQMS as an intercalator in the redox signal enhancement through inserting between the double-helix structures of the dsDNA based on the help of its planar aromatic ring. Moreover, no DNA peak absorption at 225 nm, which indicated that the DNA was hybridized successfully.

**Scheme 2:**
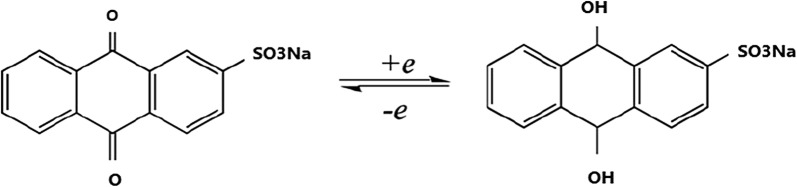
The proposed mechanism for electrochemical reaction of AQMS

The oxidation and reduction products of two hydrogen ions and two electron transfers from AQMS reagent can easily carry throughout the elongated and rigid structure of double-stranded DNA which can be measure as a current charge by DPV method [35, 36] (Scheme 2).

The electrodynamic characteristics of modified SPCE with l-cysteine-AuNPs and rGO-MWCNTs were obtained by means of differential pulls voltammetric method in PBS containing AQMS. The potential difference of oxidation/reduction of AQMS (Ep) and electrical current flow (i_p_) were determined based on Nicholson-Shain and Randles–Sevcik equations, respectively as follows [37]:

3 $$E_{P} = E^{o} - \left( {\frac{RT}{3nF}} \right)\ln \left[ {\frac{{4.78\varPi 3D_{O} }}{{2D_{R} }}} \right] - \left( {\frac{RT}{3nF}} \right)\ln \left( {\frac{\alpha }{{kC_{O}^{*} }}} \right)$$

4 $$i_{p} = (2.69 \times 10^{5} )n^{{{3 \mathord{\left/ {\vphantom {3 2}} \right. \kern-0pt} 2}}} .A.C^{*} .D^{{{1 \mathord{\left/ {\vphantom {1 2}} \right. \kern-0pt} 2}}} .v^{{{1 \mathord{\left/ {\vphantom {1 2}} \right. \kern-0pt} 2}}}$$

Where, k is the electron transfer rate constant, C^*^ is the bulk concentration of reductive/oxidative species, ʋ is the scan rate, D_R_ and D_O_ are the diffusion coefficient of the reduced and oxidized species, respectively.

As it is obvious in several reports, a sharp current peak at ≈ − 0.43 V resulted from measuring the DPV response of proposed HPV 18 biosensor was noticed.

**Fig. 4:**
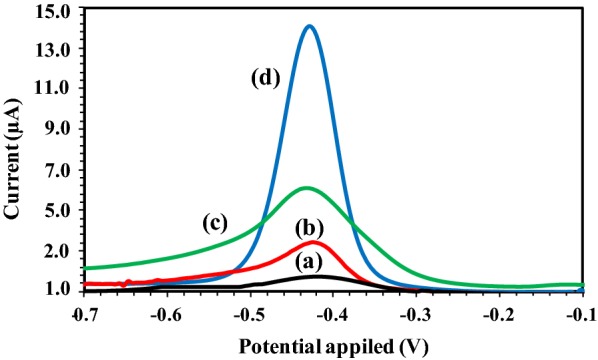
Differential pulse voltammograms for the AQMS electrochemical oxidation of bare SPCE (a), a modified electrode with l-cysteine-AuNPs (b), a modified electrode with l-cysteine-AuNPs and rGO (c) and a modified electrode with l-cysteine-AuNPs and rGO-MWCNTs (d). The DPV analysis was conducted in 0.05 M PBS (PH 6.5) with a scan rate of 50 mV s^−1^ versus Ag/AgCl reference electrode

Figure 4 shows the DPV voltammograms for the AQMS electrochemical oxidation of bare SPCE (a), modified electrode with l-cysteine-AuNPs (b), modified electrode with l-cysteine-AuNPs and rGO (c), modified electrode with l-cysteine-AuNPs and rGO-MWCNTs (d) in 0.05 M PBS (pH 6.5) with a scan rate of 50 mV s^−1^. As seen in Fig. 4, it was observed that the bare SPCE showed the lowest current peak which represents a poor electron transfer [19]. In contrast, modifying the SPCE with l-cysteine-AuNPs showed a slight increase in the current signal rate of about 2 μA due to AQMS oxidation at SPCE-solution interface via offering catalytic properties and excellent conductivity of AuNPs [38]. The highest current peak is related to SPCE modified with l-cysteine-AuNPs and rGO-MWCNTs. This is confirmed that rGO-MWCNT nanocomposite has a remarkable effect on the sensitivity of the modified electrode due to its excellent conductivity and high surface-to-volume ratio via promoting and possessing the electron transfer and rapid electrode kinetics, respectively [39, 40]. In addition, the obtained results proved that the AuNPs as excellent electric conductive materials provide a suitable surface for immobilization of thiolated molecules through chemisorptions. The electric conductive molecules such as MWCNTs and rGO containing both thiol (supplied by l-cysteine) and carboxyl groups can be used as linkers for functionalized AuNPs. The solubility of rGO in aqueous solutions provides its wide application in biological samples.

### Optimization and characterization of HPV 18 DNA biosensor

#### Effect of DNA probe

**Fig. 5:**
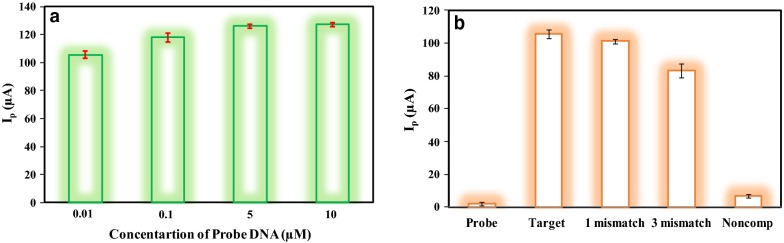
Histogram of (**a**) the HPV18 probe concentration effect on the AQMS electrochemical oxidation during immobilization process in 0.05 M PBS pH 7. The electrochemical analysis was studied by DPV method in the following conditions: initial potential − 0.7 V, end potential − 0.2 V, modulation amplitude 50 mV and scan rate 50 mVs^−1^. All results were performed with triplicate measurement of each HPV18 probe concentration, and (**b**) DPV oxidation current signal of HPV-ssDNA immobilized at the surface of SPCE during hybridization with DNA Target, DNA one mismatch, DNA three mismatch and non-complimentary DNA in 0.05 M PBS at pH 7 with the scan rate of 50 mVs^−1^

In this study, the electrochemical behavior of the probe concentration on the modified SPCE through AQMS electrochemical oxidation was investigated. As shown in Fig. 5a, the effect of probe concentration was obtained by using DPV technique in 0.05 M PBS (pH = 7). The results demonstrated that the concentrations of 0.01 μM and 0.1 μM indicate the lowest signals due to the low amount of DNA probe immobilized on the surface of modified SPCE. The concentration of 5 μM showed the highest current peak which resulted in the most hybridization of target cDNA. Also, no increase in signal strength was observed for a concentration of 10 μM or higher. It can be explained by the lower availability of the intercalator AQMS and massive probe accumulation on the surface of SPCE that causes probe overloading. As a result, the concentration of 5 μM was determined as the optimal concentration for DNA probes [41, 42].

#### Effect of hybridization for synthetic HPV 18 DNA target

The DNA target was detected through the DPV responses of the modified biosensor to the electrochemical oxidation of AQMS. For determining the selectivity of biosensor response, the DNA probe hybridization with nil DNA target, DNA target, DNA one mismatch, DNA three mismatch and DNA non-complementary was investigated. In Fig. 5b, the highest current peak is related to the hybridization of the probe-modified electrode and cDNA but unlike that, the lower current is due to the use of non-complementary DNA. Also, the use of one mismatch DNA and three mismatch DNA results in lower signal flow, due to the lack of complete hybridization. This can be explained due to more AQMS accumulation on the ds-DNA caused by the affinity of AQMS to the double-helix structure of the dsDNA.

### Calibration curve

**Fig. 6:**
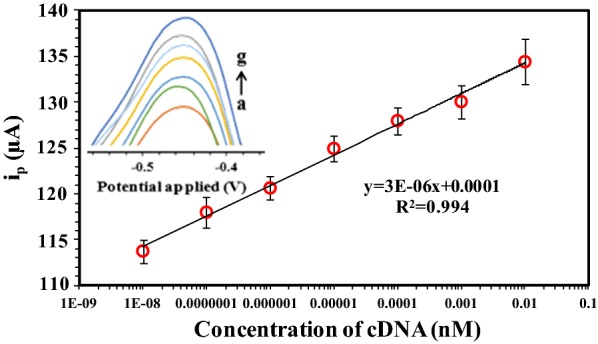
The differential pulse voltammograms and calibration plots for the AQMS electrochemical reduction after hybridization with different concentration of DNA target (a: 10^−8^, b: 10^−7^, c: 10^−6^, d: 10^−5^, e: 10^−4^, f: 10^−3^ and g: 10^−2^ nM) in 0.05 M PBS pH 7. The DPV method was applied under the following conditions: initial potential − 0.7 V, end potential − 0.2 V, modulation amplitude 50 mV and scan rate 50 mVs^−1^

To identify the conditions of optimized HPV-18 DNA biosensor, the oxidation of AQMS was measured during hybridization of DNA probes with different concentrations of synthetic DNA targets (0.01fM to 0.01 nM) by the DPV method. Figure 6 shows that with increasing concentration of DNA target, an increasing trend in the current signal was observed. It is thus believed that by increasing the concentration of DNA target and subsequently increasing their hybridization with the DNA probe, AQMS reduction increases peak current. A calibration curve of the current signals (ΔI) various the concentrations of targets DNA in from the DPV analysis was plotted. As it is obvious from Fig. 6, the calibration curve exhibited a linear dynamic range in a concentration range of 0.01 fM to 0.01 nM with a correlation coefficient of 0.994. Moreover, the DPV peak potential shows a slightly negative shifts by about 10 mV with the increase of the DNA target concentration in the studied concentration range. Presumably, this shift is due to the Donnan equilibria in the nanocomposite film [43, 44].

The derived limit of quantitation (LOQ) (at 10SD/α) and limit of detection (LOD) (at 3SD/α) [16, 45] of the proposed HPV-18 DNA biosensor were calculated to be 0.16 and 0.05 fM, respectively. In these equations, SD is defined as the standard deviation of the intercept and α is defined as the slope of the linear regression. The stability and repeatability of the proposed biosensor were also examined. The relative standard deviation of the five repetitive measurements was 1.30%, which is less than 20%, demonstrating the good reusability and stability of the proposed biosensor.

### FTIR-ATR analysis

**Fig. 7:**
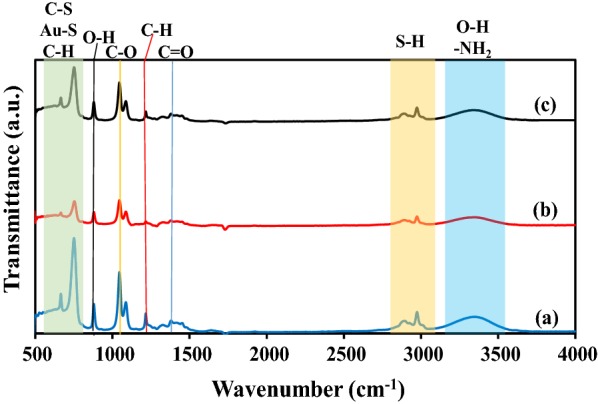
FT-IR spectra of (a) modified SPCE with l-cysteine-AuNPs and rGO-MWCNTs (b) ssDNA HPV-18 probe immobilized SPCE and (c) ssDNA hybridized SPCE

The FTIR-ATR spectra of functional groups of modified SPCE with (a) l-cysteine-AuNPs and rGO-MWCNTs (b) ssDNA probe and (c) dsDNA exhibits a peak series in the region of 4000–500 cm^−1^ (Fig. 7). The FTIR-ATR spectra of rGO-MWCNT/L-Cys-AuNPs composite shows important peaks at 3370 cm^−1^, 2974 cm^−1^, 1452 cm^−1^ and 750-665 cm^−1^ for (O–H stretch), (S–H stretch), (C = O stretch) and (C-S; Au–S stretches), respectively (a). The formation of self-assembled monolayer modified SPCE (l-Cys/Au SAMs) was obtained through combining the l-Cys onto AuNPs by taking advantage of strong Au–S interaction. The immobilization of ssDNA probe onto a modified SPCE with rGO-MWCNT/l-Cys-AuNPs lead to a decrease in peak intensity related to Au–S, C–S and O–H stretching (b). A FT-IR spectrum of ssDNA HPV-18 after hybridization with genomic DNA exhibits similar peaks of different bases at the same position with an increase in peak intensity (c). The N–H bending and stretching of purine and pyrimidine ring as DNA bases, is corresponded to the presented peaks at 3355 cm^−1^ [35]. The presented results show the successful process of immobilization and hybridization of the ssDNA probe with genomic DNA.

### FESEM analysis

**Fig. 8:**
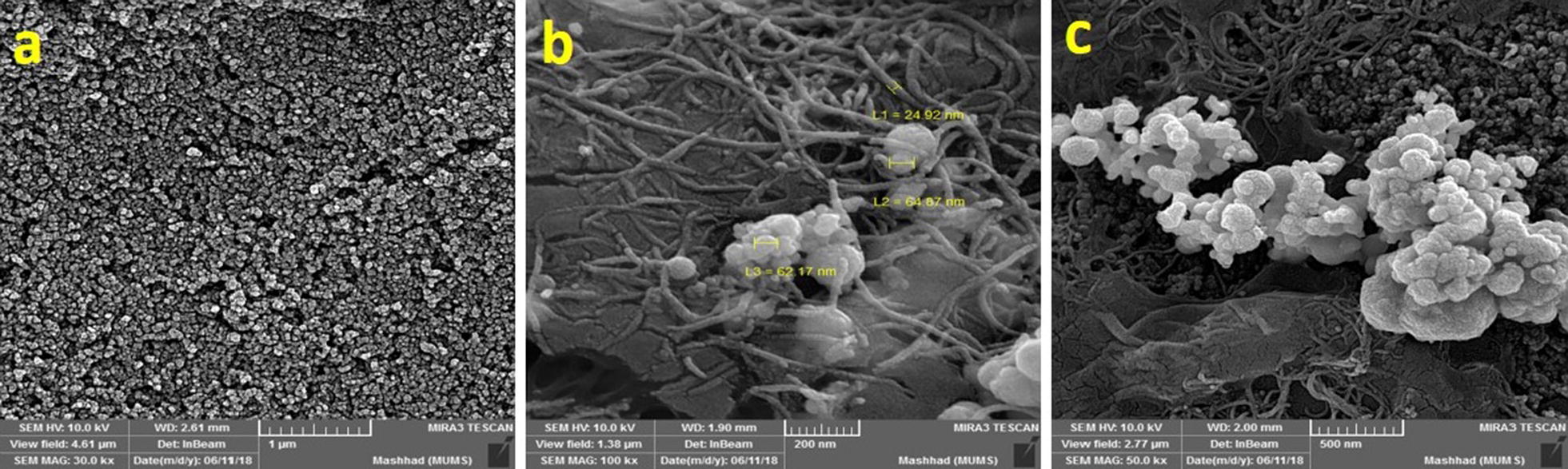
FESEM image of (**a**) bare SPCE (scale 1000 nm), (**b**) modified SPCE with rGO-MWCNTs/AuNPs (scale 200 nm) and (**c**) modified SPCE with rGO-MWCNTs/AuNPs-l-cysteine (scale 500 nm)

To confirm the rGO-MWCNT/AuNPs composites stabilization on the surface of SPCE, the field-emission scanning electron microscopy (FESEM) examination was used [12]. Figure 8a, b, and c are FESEM nano-graphs of the bare SPCE, rGO-MWCNT/AuNPs composite modified SPCE with and without l-cysteine, respectively. As it is shown in Fig. 8a, the graphitic carbon powder is responsible for the small particles dispersed throughout the bare SPCE which assigned the sheet-like structures. After modifying the SPCE with rGO-MWCNT composite, the presence of MWCNTs which bridge and covered the rGO sheets and small particles of graphitic powder were observed. The presence of AuNps which improves the electrocatalytic activity by enhancing the conductivity for fast electron transfer, is seen that distributed on the rGO-MWCNT network in Fig. 8b, c. Moreover, as shown in Fig. 8b, when AuNPs are deposited on the SPCE surface modified with rGO-MWCNT, a partial accumulation of them is observed which is partly due to the strong Van der Waals force between the nanomaterial. In the following, after electrodeposition of l-cysteine on the modified SPCE, the accumulation of AuNPs decreases and the particle size increases (Fig. 8c) [24].

### EIS analysis

**Fig. 9:**
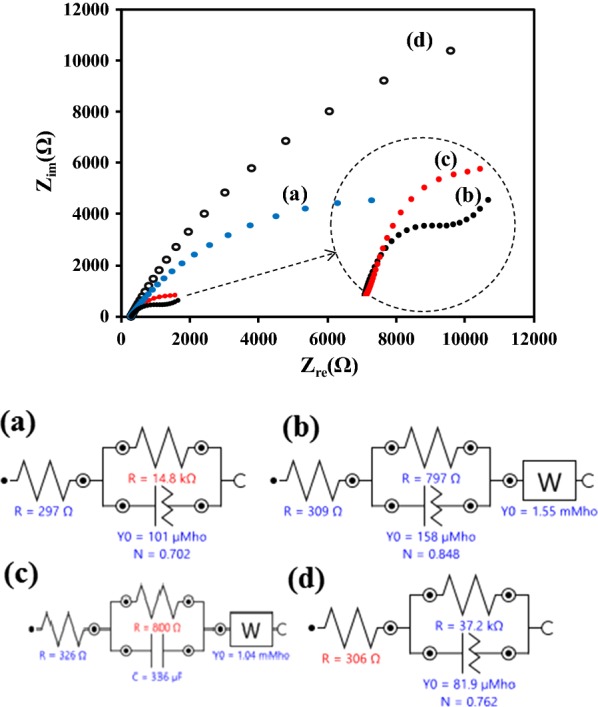
Nyquist plots and equivalent circuit models of the impedance spectra recorded for SPCE modified with (**a**) rGO-MWCNTs, (**b**) rGO-MWCNTs/AuNPs, (**c**) rGO-MWCNTs/AuNPs-l-cysteine-ssDNA probe and (**d**) rGO-MWCNTs/AuNPs-l-cysteine-ssDNA probe with DNA target

In this section, the EIS was applied as an effective technique for investigation of the proposed electrode behavior after each assembly steps. The EIS recorded in 1 × 10^−3^ M [Fe(CN)6]^−3^/[Fe(CN)6]^−4^ (1:1) containing 0.1 M KCl solution in the frequency range of 0.1–100 kHz followed by applying a signal amplitude of the open circuit potential. As it is obvious in Fig. 9, the Nyquist plots of SPCE modified with (a) rGO-MWCNTs, (b) rGO-MWCNTs/AuNPs, (c) rGO-MWCNTs/AuNPs-l-cysteine-ssDNA probe and (d) rGO-MWCNTs/AuNPs-l-cysteine-ssDNA probe with DNA target. The SPCE modified with rGO-MWCNTs showed a resistance (14.8 kΩ), which indicated that rGO-MWCNTs was an electric conducting material and accelerated the electron transfer. The Rct decreased evidently (797 Ω) because AuNPs loaded on the surface of SPCE/rGO-MWCNTs modified electrode formed an excellent carrier and accelerated the further electron transfer. The loading of the ssDNA probe on the modified SPCE led to the enhancement in Rct up to 800 Ω. Significant enhancement Rct (37.2 kΩ) by hybridization of ssDNA probe with ssDNA target formed a barrier and further prevented the redox probe to the electrode surface (Fig. 9).

### Performance of proposed biosensor in the analysis of HPV in real sample

**Fig. 10:**
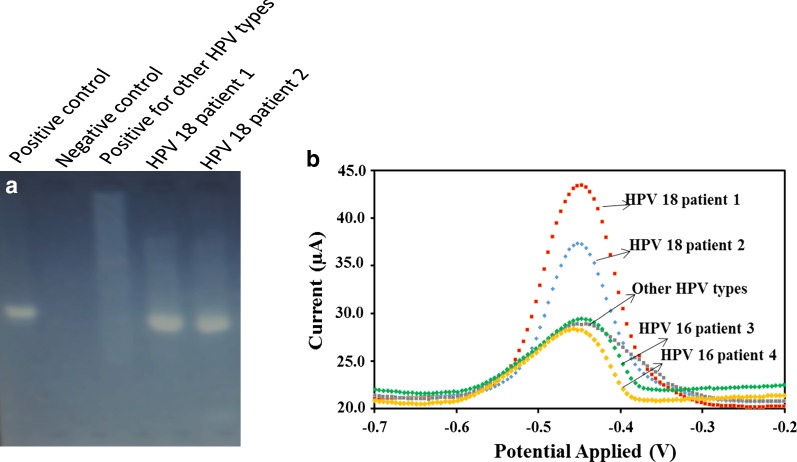
**a** DNA extracted from clinical sample and presence of DNA of HPV type 18 virus on gel electrophoresis; **b** the differential pulse voltammograms for the AQMS electrochemical reduction after hybridization with DNA extract from the real sample. The electrochemical analysis was studied by DPV method in the following conditions: initial potential − 0.7 V, end potential − 0.1 V, modulation amplitude 50 mV and scan rate 50 mVs^−1^

The performance of proposed HPV-18 DNA biosensor was investigated via applying it in the extracted DNA samples from human patient. For this purpose, the PCR was performed and the amplification products were run in the gel electrophoresis [46, 47]. As shown in Fig. 10, the results obtained from the positive DNA HPV-18 patient samples extracted from the real samples were consistent with the results of synthesized DNA. An increment in signal strength was observed with increasing target DNA concentration. In addition, the DPV signal of hybridization of the probe modified-SPCE with negative HPV-18 patient sample decreased in compare to the positive forms, indicating that there is a poor hybridization signal in the negative patient sample.

These data demonstrated that the proposed HPV-18 DNA electrochemical biosensor able to detect the HPV type 18 in PCR real samples.

Standard synthesized target ssDNA HPV18 has been spiked into the negative cervical samples within the calibration concentration range of the biosensor for recovery of target ssDNA HPV18 in the cervical samples, and 0.05 M PBS (pH 7.0) was used to stabilize the pH level of the samples. From the results tabulated in Table 3, satisfactory recoveries between 101.8 and 128% were obtained through applying the proposed DNA biosensor for accurate and reliable target DNA HPV18 determination in the cervical samples.

**Table 3:** Recovery of synthesized target DNA HPV18 in the real samples

Synthesized target DNA HPV18 concentration spiked into the negative cervical samples (fM)	Synthesized target DNA HPV18 concentration determined by DNA biosensor (fM)	Recovery (%)
0.05	0.064	128
0.1	0.121	121
1	1.15	115
20	20.5	102.5
50	57	114
250	268	107.2
500	519	103.8
1000	1018	101.8

### Comparison of proposed HPV-18 DNA biosensor with other studies

The analytical performance of current biosensor was compared with previous published HPV-18 DNA electrochemical biosensors and the results were listed in Table 4. The proposed DNA biosensor based on a SPCE modified with a nanocomposite of rGO-MWCNT/AuNPs showed an improvement in the linearity response range in compare to previous works. It is also compared favorably with other reported HPV-18 DNA biosensors in terms of the lower detection limit. Therefore, rGO-MWCNT/AuNPs modified SPE was confirmed to be an efficient platform for the electrochemical sensor. It might be mainly ascribed to the large specific surface area of the rGO-MWCNT/AuNPs nanocomposite that improved the immobilization amount of ssDNA probes and the fine electronic conduction ability of AQMS that improved the detection signals [13]. Moreover, the formation of self-assembled monolayer modified SPCE (l-Cys/Au SAMs) enhanced the electrochemical responses towards DNA targets and also provides the less prone of DNA probe to nonspecific adsorption onto the SPE surface [48]. A detailed comparison of electrochemical HPV-18 DNA biosensor reported in the literature is shown in Table 4.

**Table 4:** Comparison of HPV-18 DNA electrochemical biosensor with other previous reported electrochemical HPV-18 DNA biosensors

Ref.	Method	Sensor platform	Label	Detection limit	Detection range
[49]	CV	Gold surface/oligoethylene glycol-terminated bipodal thiol	HRP	170 pM	0.1 nM–50 nM
[50]	SWV	GCE/car-boxyphenyl layer	Hg(II)	1.2·10–5 nM	–
This study	DPV	SPE/rGO,MWCNT, Au nanoparticle, L-cysteine	AQMS	0.05 fM	0.01 fM–0.01 nM

*SWV* square wave voltammetry, *HRP* horseradish peroxide, *AQMS* anthraquninone-2-sulfonic acid monohydrate sodium salt

## Conclusion

In this study, we designed an electrochemical biosensor to detect HPV-18, one of the most hazardous types of HPVs. The performance of this biosensor was evaluated using synthetic DNA and DNA extracted from HPV 18 patients. The proposed biosensor demonstrated successful performance in identifying the HPV extracted from real samples. The biosensor was also able to detect very small quantities and a large range of analytes due to the use of rGO/MWCNT, Au nanoparticle, l-cysteine to modify the surface of the electrode. The specific response of the biosensor was examined by a test and showed a very good result. Therefore, the designed biosensor can be used as a successful tool for rapid, accurate, easy, and early detection of HPV 18 and alternative traditional and current methods.

## Data Availability

The datasets used and/or analyzed during the current study are available from the corresponding author on reasonable request.

## Abbreviations

HPV: human papillomavirus
MWCNTs: multiwalled carbon nanotubes
SPCE: screen-printed carbon electrode
AuNP: Au nanoparticle
ssDNA: single strand DNA
Cys: l-cysteine
DPV: differential pulse voltammetry
AQMS: anthraquninone-2-sulfonic acid monohydrate sodium salt
PCR: polymerase chain reaction
EIS: electrochemical impedance spectroscopy
LCD: liquid–crystal displayer
CV: cyclic voltammetry
LOQ: limit of quantitation
LOD: limit of detection
FESEM: field-emission scanning electron microscopy
SWV: square wave voltammetry
HRP: horseradish peroxide
